# Supplementary material for: Relationship between Alcohol Intake and Chronic Pain with Depressive Symptoms: A Cross-Sectional Analysis of the Shika Study
Source: Int J Environ Res Public Health. 2022 Feb 11;19(4):2024. doi: 10.3390/ijerph19042024 (PMC8871655; doi:10.3390/ijerph19042024)
Supplement: Supplementary file 1 [file ijerph-19-02024-s001.zip › ijerph-1559111-supplementary.pdf]

Table S1. Differences between participants with and without depressive symptoms in females

	Without depressive symptoms (n = 667)		With depressive symptoms (n = 350)		p-Value [a]
	Mean / n	SD / %	Mean / n	SD / %	
Age, years	68.80	8.46	71.39	10.78	**<0.001**
Living alone, n	98	11.95	52	13.13	0.558
Education, years	10.99	2.46	10.65	2.57	**0.025**
Without exercise/hobbies, n	477	85.17	303	76.52	**<0.001**
Smoking history, n	31	3.78	13	3.28	0.663
BMI, kg/m^2	22.78	3.08	22.36	3.50	**0.040**
Diabetes treatment, n	60	7.32	33	8.33	0.532
Hyperlipidemia treatment, n	168	20.49	50	12.63	**<0.001**
Hypertension treatment, n	252	30.73	120	30.30	0.879
Alcohol (crude data), g	2.05	6.01	1.96	6.86	0.829
Alcohol (density method), % energy	0.52	1.51	0.49	1.63	0.794
No alcohol intake, n	603	73.54	305	77.02	0.191
CP					
Any, n	66	8.05	65	16.41	**<0.001**
Head, n	1	0.12	3	0.76	0.104
Neck/shoulder/upper limb, n	25	3.05	21	5.30	0.053
Low back/knee, n	49	5.98	46	11.62	**<0.001**
Foot, n	12	1.46	11	2.78	0.115

Notes: [a] p-values were calculated using the Student's t-test for continuous variables and the chi-squared test for categorical variables (p-values less than 0.05 are highlighted in bold). **Abbreviations:** SD, standard deviation; BMI, body mass index; CP, chronic pain.

Table S2. Differences between chronic pain groups stratified in females

	Non-CP (any) (n = 1085)		CP (any) (n = 131)		p-Value [a]
	Mean / n	SD / %	Mean / n	SD / %	
Age, years	69.23	9.13	73.08	10.45	**<0.001**
Living alone, n	128	11.80	22	16.79	0.100
Education, years	10.96	2.48	10.22	2.60	**0.002**
Without exercise/hobbies, n	687	63.32	93	70.99	0.084
Smoking history, n	38	3.50	6	4.58	0.533
BMI, kg/m^2	22.63	3.20	22.77	3.42	0.650
Diabetes treatment, n	82	7.56	11	8.40	0.733
Hyperlipidemia treatment, n	204	18.80	14	10.69	**0.022**
Hypertension treatment, n	335	30.88	37	28.24	**0.537**
Depressive symptoms, n	331	30.51	65	49.62	**<0.001**
Alcohol (crude data), g	2.09	6.42	1.45	5.16	0.199
Alcohol (density method), % energy	0.53	1.57	0.37	1.30	0.197
No alcohol intake, n	801	73.82	107	81.68	0.051
CP					
Head, n	0	–	4	3.05	–
Neck/shoulder/upper limb, n	0	–	46	35.11	–

Low back/knee, *n*	0	–	95	72.52	–
Foot, *n*	0	–	23	17.56	–

Notes: [a] *p*-values were calculated using the Student's *t*-test for continuous variables and the chi-squared test for categorical variables (*p*-values less than 0.05 are highlighted in bold). **Abbreviations:** SD, standard deviation; BMI, body mass index; CP, chronic pain.

Table S3. Interactions between depressive symptoms and CP on alcohol intake (density method) in females

		Non-CP (any) (*n* = 1085)		CP (any) (*n* = 131)		DS	CP	DS × CP
		Mean (95%CI)	*n*	Mean (95%CI)	*n*			
Head pain	NDS	0.50 (0.40, 0.61)	819	-0.21 (-3.19, 2.78)	1	0.979	0.391	0.958
	DS	0.53 (0.37, 0.68)	393	-0.27 (-2.00, 1.45)	3			
Neck/shoulder/ upper limb pain	NDS	0.50 (0.40, 0.61)	795	0.51 (-0.09, 1.10)	25	0.937	0.983	0.998
	DS	0.52 (0.36, 0.68)	375	0.52 (-0.13, 1.18)	21			
Low back/knee pain	NDS	0.52 (0.41, 0.62)	771	0.30 (-0.13, 0.73)	49	0.523	0.497	0.538
	DS	0.52 (0.36, 0.68)	350	0.51 (0.06, 0.95)	46			
Foot pain	NDS	0.50 (0.40, 0.61)	808	0.57 (-0.30, 1.43)	12	0.603	0.688	0.544
	DS	0.53 (0.37, 0.68)	385	0.21 (-0.69, 1.10)	11			

Notes: [a] Analysis of covariance (p-values less than 0.05 are highlighted in bold). Adjusted for BMI, age, living alone, education, without exercise/hobbies, smoking history, BMI, diabetes treatment, hyperlipidemia treatment, and hypertension treatment.
Abbreviations: CP, chronic pain; NDS, no depressive symptoms; DS, depressive symptoms; CI, confidence interval; BMI, body mass index.

Table S4 Relationship between alcohol intake and CP stratified by depressive symptoms in females

		Exp(B)	95%Cl Lower	96%Cl Upper	*p*-Value
With depressive symptoms (n = 396)	Alcohol (density method)	1.017	0.861	12.010	0.842
Without depressive symptoms (n = 820)	Alcohol (density method)	0.871	0.672	1.130	0.300

Notes: Significant estimates are in bold. Adjusted for BMI, age, living alone, education, without exercise/hobbies, smoking history, BMI, diabetes treatment, hyperlipidemia treatment, and hypertension treatment. **Abbreviations:** Exp (B), Exponentiation of the B coefficient; CI, confidence interval.
